# Cisplatin or Not in Advanced Gastric Cancer: A Systematic Review and Meta-Analysis

**DOI:** 10.1371/journal.pone.0083022

**Published:** 2013-12-27

**Authors:** Fausto Petrelli, Alberto Zaniboni, Andrea Coinu, Mary Cabiddu, Mara Ghilardi, Giovanni Sgroi, Sandro Barni

**Affiliations:** 1 Medical Oncology Unit, Oncology Department, Azienda Ospedaliera Treviglio, Treviglio (BG), Italy; 2 Medical Oncology Unit, Istituto Ospedaliero Fondazione Poliambulanza, Brescia, Italy; 3 Surgical Oncology Unit, Surgery Department, Azienda Ospedaliera Treviglio, Treviglio (BG), Italy; University General Hospital of Heraklion and Laboratory of Tumor Cell Biology, School of Medicine, University of Crete, Greece

## Abstract

**Background:**

Cisplatin-based chemotherapy is frequently used to treat advanced gastric cancer (GC). Although it leads to increased overall survival (OS) when added to single agents or chemotherapy doublets, toxicity is also generally increased. The purpose of this meta-analysis study was to compare the efficacy of fchemotherapy with and without cisplatin in patients with advanced GC.

**Methods:**

Randomised trials that compared first-line cisplatin-based chemotherapy with regimens in which cisplatin was replaced by other agents were identified by electronic searches of PubMed, EMBASE, the Web of Science, and the Cochrane Central Register of Controlled Trials. Meta-analysis was performed using a fixed or random effects model. OS, reported as a hazard ratio (HR) and a 95% confidence interval (CI), was the primary outcome measure.

**Results:**

Fourteen trials (5 phase III and 9 phase II), including 2,981 patients, were identified. Overall, chemotherapy regimens without cisplatin significantly improved OS (HR, 0.79; 95% CI, 0.68–0.92; *p* = 0.003), progression-free survival (PFS) (HR, 0.77; 95% CI, 0.66–0.90; *p* = 0.001), and response rate (RR) (OR, 1.25; *p* = 0.004) when compared to cisplatin-containing regimens. A subgroup analysis according to histology, site of the primary tumour and extent of disease was not possible due to lack of data.

**Conclusions:**

Compared with cisplatin-based doublets and triplets, combinations in which cisplatin was replaced by new drugs improved outcome and RRs in randomised trials for advanced GC and therefore should be strongly considered in the metastatic setting. A limitation of this meta-analysis is that we cannot identify a subgroup of patients (according to histology, site of primary tumour or burden of metastatic disease) which could derive greater benefit from cisplatin-free chemotherapy.

## Introduction

Palliative chemotherapy, which is usually 5-fluorouracil (5-FU) based, is the main treatment for advanced (metastatic or unresectable) gastric cancer (GC); it has been shown to increase the median overall survival (OS) from 4.3 to 11 months as compared with the best supportive care [1]. Doublet and triplet combinations (5-FU and cisplatin [CDDP] based) have resulted in more modest increases in the median time to progression (TTP) and OS (about 2 and 1.5 months, respectively), but they have increased toxicity compared to single agents. The addition of CDDP to the 5-FU/anthracycline doublet resulted in a significant increase in OS compared to 5-FU/anthracycline alone (hazard ratio [HR], 0.82; 95% confidence interval [CI], 0.73–0.92) [1]. Recently, an individual patient data meta-analysis of 22 trials was performed by the Global Advanced/Adjuvant Stomach Tumor Research International Collaboration (GASTRIC) Group. They found that the addition of any new chemotherapeutic agent to a standard control regimen resulted in slight improvements in OS and progression-free survival (PFS) [2]. In particular, an analysis of 2,337 patients from 8 trials with platinum agents showed that these agents significantly increased PFS (HR, 0.88; 95% CI, 0.81–0.96) but not OS (HR, 0.96; 95% CI, 0.88–1.05). Similar results were observed in trials of irinotecan (CPT-11), whereas docetaxel- and anthracycline-based regimens did not enhance either PFS or OS compared with the control arms.

A meta-analysis of three randomised trials showed that oxaliplatin, a platinum-based analogue of CDDP, was associated with better OS and reduced rates of neutropenia and thromboembolic events when compared to CDDP-containing regimens [3]. Moreover, an updated meta-analysis of CPT-11–based randomized trials found that CPT-11–containing regimens significantly improved outcome compared with the control arms [4].

Part of the GASTRIC meta-analysis included comparisons of control regimens with those in which one drug was replaced by an experimental agent. Nine trials were included, with new agents showing an overall HR for death of 0.85 compared with standard agents. Of these trials, however, only one compared a CDDP-based regimen with the same regimen containing a modern agent. This trial found that the modern agent did not significantly improve OS or TTP [5].

The REAL-2 study [6] demonstrated the equivalence of the CDDP and oxaliplatin arms, as well as the potential vascular toxicity of CDDP [7]; this led to the formation of the hypothesis that the replacement of CDDP by a modern drug in a doublet (or triplet) combination would result in similar efficacy outcomes and reduced toxicity. In the REAL-2 study, the oxaliplatin arms (EOF and EOX) were found to not be inferior to the CDDP arms (ECF and ECX), with the former showing a lower rate of elevated creatinine levels and thromboembolic events (7.6% versus 15.1%). Among the modern agents, the taxanes (e.g., docetaxel), the oral fluoropyrimidine capecitabine, and S-1 retained significant activity in patients with metastatic GC [8]–[10].

We have therefore performed a systematic review and meta-analysis of randomised phase II and III treatment trials that compared the efficacy of two- and three-drug regimens containing CDDP (control arms) with the same regimens in which modern agents were substituted for CDDP (experimental arms) in patients with advanced GC.

## Methods

### Search strategy and study selection criteria

Trials were identified by conducting electronic searches of the Cochrane Controlled Trials Register, PubMed, the Web of Science, and EMBASE. In addition, we manually searched reference lists and conference proceedings of the American Society for Clinical Oncology (without date limitations). The search strategy included the following terms: ‘cisplatin’ in combination with ‘stomach neoplasm’, ‘gastric cancer’, ‘gastric carcinoma’, ‘gastro-oesophageal cancer’, ‘gastro-oesophageal carcinoma’, ‘oesophago-gastric cancer’, and ‘oesophago-gastric carcinoma’ and ‘metastatic’, ‘advanced’, ‘unresectable’, ‘recurrent’, ‘stage IV’, ‘relapsed’. Only publications that were written in English and that involved humans were considered. The temporal limits searches were from inception to 16 February 2013. Abstracts of major cancer conferences were included if the information was presented on at least one of the endpoints. Studies were selected if they were randomised controlled phase II to III trials; if they included patients with histologically confirmed, advanced, recurrent, or metastatic adenocarcinoma of the stomach or gastro-oesophageal junction; and if they compared (first-line) chemotherapy with at least two or three agents, including CDDP, with chemotherapy that included the same number of cytotoxic agents and a modern agent (e.g., a taxane, CPT-11, oral fluoropyrimidine, or oxaliplatin) in place of CDDP.

Trials including oesophageal or upper gastrointestinal cancer patients were included if sufficient information was available for the subgroup of patients with GC. Trials incorporating targeted therapies, intraperitoneal chemotherapy, or radiotherapy were excluded.

Study selection, data extraction, and data entry were performed by two authors independently (FP and AC). Differences were resolved by consensus with a third author (SB).

The following information was extracted from each article: 1) basic information including journal, year of publication, and author names; 2) demographic characteristics of the patients, including median age and sex distribution; 3) study information, including sample size, study design, and study endpoints; 4) treatment information (including treatment regimens); and 5) outcomes, including median OS and median PFS (or TTP).

### Statistical analysis

OS was the primary outcome measure, and PFS (or TTP) and the response rate (RR) were the secondary endpoints. HRs and 95% CIs were estimated directly or indirectly from the reported data. When HR and 95% CI were not reported in a publication, they were computed from the other available data (Kaplan-Meier curves or number of events) [11]–[13]. The quality of the publications included in the primary outcome analysis was evaluated using the Jadad scale.

A fixed effects or random effects (weighted with inverse variance) model and the Mantel-Haenszel (M-H) method were utilized to combine and weigh the individual studies. The Cochran Q test, with a predefined significance threshold of 0.1, was used to assess the statistical heterogeneity among the studies. The assumption of homogeneity was considered invalid for *p* values less than 0.1; in this case, summary estimates were reported from the random effect models. Sources of heterogeneity were determined by subgroup stratification analysis based on study characteristics such as ethnicity, type of study (phase II or phase III), and chemotherapy regimen (CDDP versus oxaliplatin, CDDP versus not oxaliplatin, CDDP versus CPT-11, and CDDP versus taxanes).

Finally, potential publication biases were evaluated using funnel plots for OS analysis, which were used to assess the relative symmetry of the individual study estimates around the overall estimate, based on Begg's and Egger's tests. A two-tailed *p* value less than 0.05 without adjustment for multiplicity was considered statistically significant. The leave-one-out procedure was also performed for primary endpoint analysis. The ‘fail-safe *N*’, which is defined as the number of additional ‘negative’ studies (studies in which the intervention effect was zero) required to increase the *p* value for the meta-analysis to above 0.05, was calculated. A two-tailed p value of less than 0.05 was considered statistically significant. Results of the meta-analysis were reported as classic forest plots (for OS and for PFS or TTP).

All statistical analyses were performed with the Review Manager 5.1 (Review Manager (RevMan) version 5.1; Copenhagen: The Nordic Cochrane Centre, The Cochrane Collaboration, 2008) and Comprehensive Meta Analysis software (version 2.2.064; July 27, 2011).

## Results

Our electronic searches revealed 2,514 references. After excluding duplicate publications and irrelevant trials, 22 references remained for further evaluation. Finally, 14 publications were included in the review [5]–[6]; ; 9 of which were reports of clinical trials in full-text publications and 5 of which were conference abstracts (Table S1). Five of the included trials were phase III, and 9 were phase II. All 14 trials included at least 1 CDDP-based control arm. The experimental arms included oxaliplatin in 5 studies, taxanes in 3, CPT-11 in 3, carboplatin/taxane in 1, S-1 in 1, and 5-FU in 1 as a replacement for CDDP. The control arms were CDDP/fluoropyrimidine based (with one also including an anthracycline) in 11 of the studies, CDDP/taxane based in 2, and CDDP/CPT-11 based in 1. One study had a 4-arm design, 1 had a 3-arm design, and 12 had a 2-arm design. Twelve trials compared chemotherapy doublets, and 2 compared chemotherapy triplets. The total number of patients randomly assigned in these trials was 2,981, with 1,501 receiving CDDP-containing chemotherapy and 1,480 receiving non-CDDP-containing chemotherapy. A consort diagram of the stepwise identification of eligible studies is detailed in Fig. S1.

### Primary endpoint: OS

Nine of the 14 trials [5−6]; reported OS data in the form of HRs or these ratios were calculated from the published data. In particular, data for computing HRs were extracted from Kaplan-Meier curves in three studies [17−18], and from the number of events in each arm and the *p* value (a randomization ratio of one to one) in one study [16].

Using a random effects model, we found that, overall, CDDP-free chemotherapy significantly improved OS compared with CDDP-containing chemotherapy (HR, 0.79; 95% CI, 0.68–0.92; *p* = 0.003; Fig. S2) with moderate heterogeneity among the studies (I^2^ = 50%, *p* = 0.04). The results remained unchanged after the leave-one-out procedure.

### Secondary endpoints: PFS and RR

Nine trials [5−6]; reported HRs for PFS or provided data that allowed the HR and PFS to be calculated, with three of these trials reporting TTP instead of PFS. Overall, CDDP-free chemotherapy significantly improved PFS compared with CDDP-containing chemotherapy using the random effects model (HR, 0.77; 95% CI, 0.66–0.90; *p* = 0.001; Fig. S3), and there was moderate heterogeneity among the studies (I^2^ = 48%, *p* = 0.05).

All 14 trials [5]-[6]; reported RR data, with the pooled results using a fixed effects model showing that CDDP-free chemotherapy significantly improved the RR compared to CDDP-containing chemotherapy (OR, 1.25; 95% CI, 1.07–1.45; *p* = 0.004; Fig. S4). There was low heterogeneity among the trials (I^2^ = 16%, *p* = 0.28).

### Subgroup analysis

Trials in eastern and western countries yielded similar GCs and outcomes. Compared with the phase III trials, the phase II trials showed better RRs (OR, 1.46 versus 1.19), OS (HR, 0.65 versus 0.93), and PFS (HR, 0.68 versus 0.89). When the agents substituted for CDDP were compared, CPT-11, oxaliplatin, and taxanes yielded similar ORs for the RR (1.26, 1.17, and 1.64, respectively), and CPT-11 and oxaliplatin yielded similar HRs for OS (0.79 and 0.83, respectively) and PFS (0.71 and 0.89, respectively); only one taxane trial reported OS and PFS; hence, formal analyses were not possible. After the exclusion of the three oxaliplatin trials, the HRs for OS and PFS remained significant in favour of experimental platinum-free trials (HR 0.77; *p* = 0.001 and HR 0.74; *p* = 0.0002). For the RR, the OR was 1.45 (*p* = 0.007).

### Publication bias

A funnel plot and both Begg's and Egger's tests were performed to assess the publication bias of the selected studies. The shapes of the funnel plots showed no evidence of obvious asymmetry (*p* = 0.348 for OS; Fig. S5). The results from Egger's test were not significant (*p* = 0.083). Using the trim-and-fill method to account for the asymmetric studies in the funnel plot had no effect on the HR for OS. The fail-safe *N* was 33, indicating that it was necessary to locate and include 33 ‘null’ studies for the combined two-tailed *p* value to exceed 0.050.

## Discussion

This meta-analysis is, to the best of our knowledge, the first to compare chemotherapy regimens with and without CDDP. The results show that replacing CDDP with oxaliplatin, CPT-11, or a taxane significantly increases OS and PFS; it also increases the RR by 25%. In particular, CDDP-free regimens have been found to reduce the risks of progression and death by 23% and 21%, respectively. The results were identical for the oxaliplatin-free trials among the experimental arms. Therefore, this meta-analysis, which analyzed five phase III trials and nine phase II trials, all except five of which were published as full texts, affirms the notion that the incorporation of modern agents other than CDDP to any chemotherapy regimen for GC significantly reduces the risk of death when compared to CDDP-based chemotherapy. Our analysis compared any two- and three-drug regimen that included CDDP with any regimen containing the same number of agents in which CDDP was replaced by oxaliplatin, CPT-11, or a taxane. It is the first analysis of its type. In contrast, the GASTRIC meta-analysis [2] included only 3 of our 14 trials. Of the 8 trials in the GASTRIC analysis that included CDDP treatment, 4 compared CDDP-based combination chemotherapy with suboptimal 5-FU alone or older regimens (FAM or ELF). Our results showed that fluoropyrimidine/oxaliplatin or CPT-11 combinations or a taxane-based regimen could actually be considered standard treatment for patients with advanced gastro-oesophageal adenocarcinomas.

Our meta-analysis has included all randomised phase II and III trials, published or not, containing modern drugs with activity in GC and has compared regimens with the same number of agents (at least doublets or triplets). We have found that patient outcomes are generally improved by the substitution of modern agents such as oxaliplatin for CDDP and by the addition of CPT-11 to established regimens. A meta-analysis of three trials comparing CDDP- with oxaliplatin-containing regimens showed that oxaliplatin significantly improved PFS (HR, 0.88; *p* = 0.02) and OS (HR, 0.88; *p* = 0.04) [3]. A meta-analysis of 10 trials of CPT-11 in Asia [4] also showed that the addition of CPT-11 to chemotherapy regimens enhanced both PFS and OS, with similar rates of hematologic and non-hematologic toxicities except for fatigue.

The renal and gastrointestinal toxicities of CDDP and the association of this agent with potential life-threatening vascular toxicities (e.g., venous thromboembolic events [7]), suggests that its current role in the treatment of advanced GC should be reassessed. Advanced GC is a lethal disease, and treatment with experimental regimens enhances OS survival by only about 3 to 4 weeks compared to standard regimens [2].

If we consider the high activity of oral agents (capecitabine and S-1) and the administration of agents such as oxaliplatin, paclitaxel, docetaxel, and irinotecan, whose infusion duration and possibly worrisome toxicities could be reduced, using cisplatin-free regimens to treat this disease could be more convenient and feasible for patients. In addition CDDP-free chemotherapy could possibly be offered to both cisplatin-fit and -unfit patients (e.g., those with poor renal function, are elderly, or have bad performance status or those who cannot tolerate forced hydration).

However, our analysis does have some limitations. First, it is a meta-analysis of published studies, with HRs for OS and PFS derived (or calculated) directly from the publications or abstracts. Thus, formal subgroup analyses, including adjusting for baseline factors such as sex, histology, site of primary disease, or extent of metastases, among the included trials was not possible. A different sensibility to citotoxic drugs has been recently suggested for intestinal and proximal subtypes of GC [Bittoni et al. in press] but not yet prospectively validated. In the trials enclosed into this meta-analysis, no analysis was possible according to histologic subtypes and sites of metastatic disease due to lack of data. In trials published in full text Second, clear differences among regimens could not be determined, although similar OS and PFS results were calculated for oxaliplatin (mainly the FOLFOX regimen) and CPT-11 (mainly CPT-11 coupled with fluoropyrimidines). Except for RRs, outcomes could not be analysed in the taxane trials due to a lack of data. Third, two-thirds of the studies included in our analysis were phase II trials and were not adequately powered for determining a survival difference or used RR as the primary endpoint. Fourth the inclusion of trials of different ethnicity could have lead to potentially mixed or even better results. Five trials included patients of Eastern countries, and they presented the highest median OS among all included trials, probably because Asiatic patients have a better prognosis, and derive the greatest benefit by oral fluropyrimidines (e.g S-1). After exclusion of two Asiatic studies in OS and PFS analysis, the results did not changed. Finally, our analysis did not evaluate the effects of treatment according to HER-2 status. In fact, the addition of trastuzumab to CDDP plus fluoropyrimidine doublets was shown to enhance survival in patients with HER-2–positive GC [26]. To our knowledge, no published randomised trial has compared chemotherapy with and without CDDP plus trastuzumab. However, data from observational practice have shown that combinations without CDDP are feasible and as active as CDDP-based regimens [27]. In the mean time, anyhow, the labelled indication of trastuzumab needs the association with CDDP and 5-FU according to the the registrative TOGA trial.

Our results, suggest for the first time that CDDP-free chemotherapy was more effective than CDDP-based chemotherapy in almost 3,000 patients with stage IV GC. According to our data, however, we cannot identify a subset of patients for whom oxaliplatin-based chemotherapy is recommended. This appears as the major weakness of this meta-analysis.

A recent phase III randomized trial comparing oxaliplatin plus capecitabine with observation as an adjuvant treatment after curative D2 surgery of GC [28] showed that an oxaliplatin-based doublet increased PFS (HR, 0.56), whereas second-line docetaxel or CPT-11, following combinations of fluoropyrimidine and platinum salts, had OS benefits. These observations, in addition to the results of this meta-analysis, confirm that CDDP may not be necessary in GC, at least in HER-2 negative setting, where trastuzumab is not indicated.

In conclusion, we found that CDDP-free combination chemotherapy, containing new active cytotoxic agents instead of CDDP, significantly enhances OS, PFS, and RR when compared with CDDP-based combination chemotherapy as first-line treatment of metastatic GC.

## Supporting Information

Figure S1 **Selection of Publications Included in the Pooled-analysis.**
(TIF)

Figure S2 **Meta-analysis of overall survival.**
(TIF)

Figure S3 **Meta-analysis of progression-free survival.**
(TIF)

Figure S4 **Meta-analysis of reponse rate.**
(TIF)

Figure S5 **Funnel plot for publication bias.**
(TIF)

Table S1 Characteristics of included trials.
(DOC)

Checklist S1 **PRISMA checklist.**
(DOC)
