# Supplementary figures and images for: Cisplatin or Not in Advanced Gastric Cancer: A Systematic Review and Meta-Analysis
Source: PLoS One. 2013 Dec 27;8(12):e83022. doi: 10.1371/journal.pone.0083022 (PMC3873906; doi:10.1371/journal.pone.0083022)

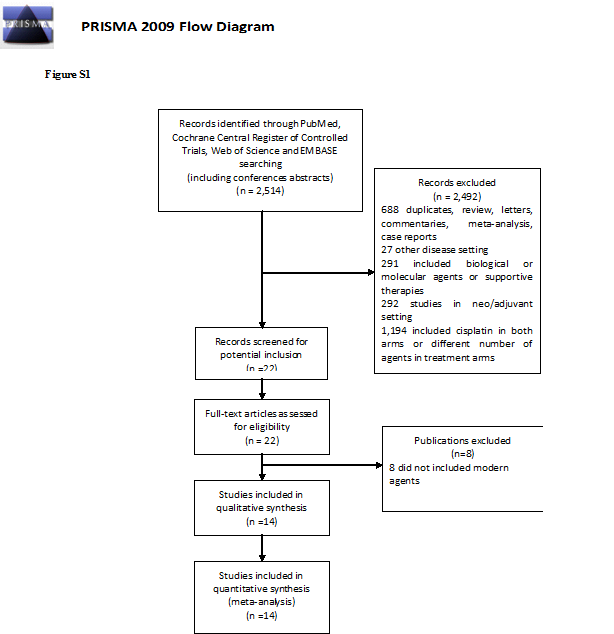

Supplement: Figure S1 — Selection of Publications Included in the Pooled-analysis. (TIF) [file pone.0083022.s001.tif]

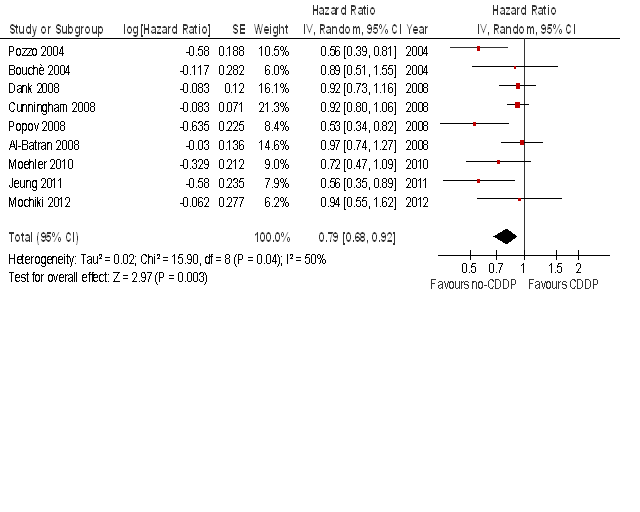

Supplement: Figure S2 — Meta-analysis of overall survival. (TIF) [file pone.0083022.s002.tif]

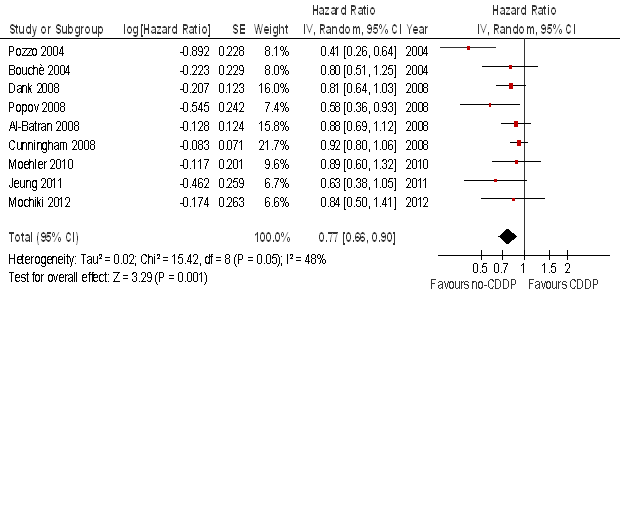

Supplement: Figure S3 — Meta-analysis of progression-free survival. (TIF) [file pone.0083022.s003.tif]

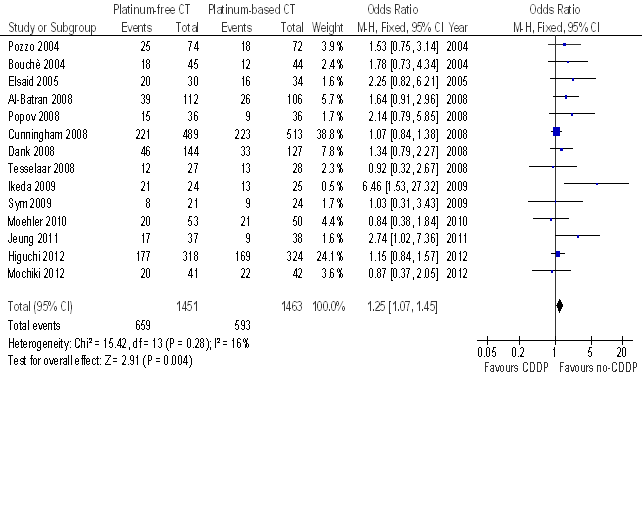

Supplement: Figure S4 — Meta-analysis of reponse rate. (TIF) [file pone.0083022.s004.tif]

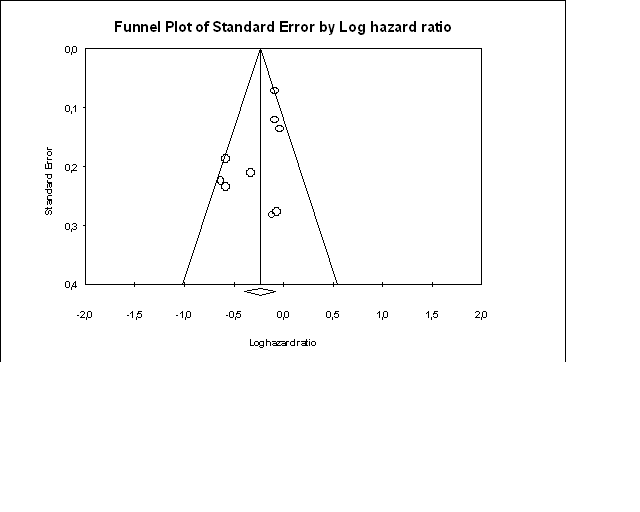

Supplement: Figure S5 — Funnel plot for publication bias. (TIF) [file pone.0083022.s005.tif]
