# Supplementary material for: Cisplatin or Not in Advanced Gastric Cancer: A Systematic Review and Meta-Analysis
Source: PLoS One. 2013 Dec 27;8(12):e83022. doi: 10.1371/journal.pone.0083022 (PMC3873906; doi:10.1371/journal.pone.0083022)
Supplement: Table S1 — Characteristics of included trials. (DOC) [file pone.0083022.s006.doc]

| **author/year** | **median age**  **(ctr vs exp)** | **type of study** | **follow up (months)** | **schedule**  **(ctr vs exp arm)** | **N° of patients** | **Primary endpoint** | **Jadad score** | **RR ctr vs exp (%)** | **PFS ctr vs exp arm (months)** | **OS ctr vs exp arm (months)** |  |
| --- | --- | --- | --- | --- | --- | --- | --- | --- | --- | --- | --- |
| **Higuchi 2012 [25]** | NA | **III** | NA | **S1/CDDP vs S1/OXA** | **642** | **PFS§/OS** | - | 52,2 vs 55,7% | 5,4 vs 5.5 | NA |  |
| **Mochiki 2012 [14]** | 63 vs 63,3 | **II** | 14 | **S1/CDDP vs S1/PAC** | **83** | RR | 2 | 48,7 vs 52,3% | 6 vs 9 | 17 vs 16 |  |
| **Jeung 2011 [15]** | 60 vs 56 | **II** | 10,2 | **DOC/CDDP vs S1/DOC** | **80** | RR | 1 | 24 vs 46% | 4.8 vs 7.3 | 8.2 vs 16 |  |
| **Moehler 2010 [16]** | 64 vs 61 | **II** | 6,1 vs 5,5  (ctr vs exp) | **X/CDDP vs X/CPT11** | **118** | RR | 1 | 42 vs 37% | 4.8 VS 4.2 | 7.9 vs 10.2 |  |
| **Sym 2009 [22]** | 50 | **II** | NA | **DOC/CDDP vs DOC/OXA** | **51** | RR | - | 37,5 vs 38,1% | 4.8 vs 4.1 | NA |  |
| **Ikeda 2009 [21]** | NA | **II** | NA | **CDDD/5FU vs S1/DOC** | **49** | RR | - | 88,3 vs 52% | 3.5 vs 6.6 | 12.36 vs 12.3 |  |
| **Popov 2008 [17]** | 57 vs 55 | **II** | 7 | **5FU/LV/CDDP vs FOLFOX** | **72** | RR and safety | 2 | 25 vs 41% | 6*vs 8* | 7 vs 10 |  |
| **Dank 2008 [5]** | 59 vs 58 | **III** | NA | **5FU/CDDP vs 5FU/CPT-11** | **333** | TTP | 3 | 25,8 vs 31,8% | 4.2* vs 5* | 8.7 vs 9 |  |
| **Al-Batran 2008 [18]** | 64 vs 64 | **III** | 14 | **5FU/LV/CDDP vs 5FU/LV/OXA** | **220** | PFS | 2 | 24,5 vs 34,8 | **3.9 vs 5.8** | 8.8 vs 10.7 |  |
| **Cunningham 2008 [6]** | 61/62 vs 65/64 | **III** | 17,5/17,6 vs 19,3/18,9  (ctr vs exp) | **ECF/ECX vs EOF/EOX** | **1,002** | OS (non inferiority) | 3 | 43,4 (n=213) vs 45,2 (n=221) | 6.2/6.7 vs 6.5/7 | 9.9/9.9 vs 9.3/11.2 |  |
| **Tesselaar 2008 [24]** | NA | **II** | NA | **weekly 5FU/LV/CDDP vs weekly 5FU/LV/PAC** | **96** | RR | - | 46 vs 44% | 3.9* vs 4.2* | 4.9 vs 5.4 |  |
| **Elsaid 2005 [23]** | NA | **III** | NA | **ECF vs DOC/CBDCA/5FU** | **64** | efficacy and tolerability | - | 47,1 vs 66,7% | NA | 8.7 vs12.4 |  |
| **Pozzo 2004 [19]** | 59 vs 57 | **II** | NA | **CDDP/CPT-11 vs 5FU/FA/CPT-11** | **115** | %CR° | 3 | 25 vs 33,8% | 4.2* vs 6.5* | 6.9 vs 10.7 |  |
| **Bouchè 2004 [20]** | 64 vs 65 | **II** | 26 | **LV-5FU2/CDDP vs LV-5FU2/CPT-11** | **134** | RR | 3 | 27 vs 40% | 4.9 vs 6.9 | 9.5 vs 11.3 |  |
| ***=time to progression; ctr=contol; exp=experimental; N°=number; RR=response rate; PFS=progression free survival; OSD=overall survival; CDDP=cisplatin; 5FU=5-Fluorouracil; LV=leucovorin; CBDCA=carboplatin; OXA=oxaliplatin; CPT-11=irinotecan; DOC=docetaxel; PAC=paclitaxel; ECF=epirubicin+cisplatin+5Fluorouracil; X=capecitabine; ECX=epirubicin+cisplatin+capecitabine; EOF=epirubicin+oxaliplatin+5Fluorouracil; EOX=epirubicin+oxaliplatin+capecitabine; FOLFOX=5Fluoruracil+leucovorin+oxaliplatin; §=noninferiority.** | | | | | | | | | | | |
